# Intimate partner violence and quality of life among mothers in Jordan during COVID-19 era

**DOI:** 10.1371/journal.pone.0298669

**Published:** 2024-04-16

**Authors:** Sawsan Abuhammad, Ahlam Al-Natour, Sarah Abu Al-Rub, Shaher Hamaideh

**Affiliations:** 1 Department of Maternal and Child Health, Faculty of Nursing, Jordan University of Science and Technology, Irbid, Jordan; 2 Department of Community and Mental Health, Faculty of Nursing, Jordan University of Science and Technology, Irbid, Jordan; 3 Faculty of Pharmacy, Jordan University of Science and Technology, Irbid, Jordan; 4 Department of Community and Mental Health Nursing, Faculty of Nursing, The Hashemite University, Zarqa, Jordan; Mutah University, JORDAN

## Abstract

**Aims:**

The study aims to examine the correlation between intimate partner violence [IPV] and quality of life [HRQoL] of mothers during COVID-19 era.

**Method:**

This study is a cross-sectional correlational study. The inclusion criteria was Jordanian women with at least 18 years old, who read and write in Arabic language and able to participate. The collection of data was done through a self-reported questionnaire distributed and completely filled with 300 married Jordanian women using social media. This study was conducted between the months of October and December 2020. The participants signed consent after being informed of their rights to exit at any point during the study and the study methods.

**Results:**

The prevalence of IPV among women was 28.3. The mean of quality of life is 86.0 [SD = 13.1) and the mean of violence is 11.9 [SD = 3.01). There was a significant negative relationship between violence and quality of life (r^2^ = .224, p = .001). This means as the violence increases, the quality-of-life decreases.

**Conclusion:**

In conclusion, there is an association between IPV and HRQoL among married people. Providing an education program and vital resources for women with the goal of preventing COVID-19 violence and assisting Jordanians become very essential.

## Introduction

Intimate partner violence (IPV) is abuse, mental, sexual, or physical, and violence by a current or previous partner IPV has a pervasive and significant threat to the health of women [1]. Mothers in all countries experienced IPV. In developed countries also mothers experience IPV. For example, three out of ten women in the US have experienced physical violence, stalking, or rape. In Jordan, five out of ten women in Jordan experienced IPV [2]. Those who experience violence by intimate partners are at a substantial risk of adverse health outcomes [2, 3]. The IPV is a source of morbidity and can cause poor life quality and mental and physical health issues [3]. Despite the availability of research in IPV, there is a need for studies using representative samples to assess IPV’s health impact accurately specifically during COVID-19 era [4]. In most cases, research that evaluates the health outcomes among women experiencing IPV often uses community-based or clinical samples [5]. Although abused women are at higher risks of experiencing poor health outcomes, it is not clear whether specific acts of violence are more injurious and have a long-lasting impact on women than others [6, 7]. Several research studies have focused on more severe types of violence, leading to less attention to minor forms of abuse [5, 6, 8].

In the past, the impact of the IPV on women’s health was given little attention in analysis [9]. Evaluating specific types of abuse and their impact on the well-being of women is essential because different acts of violence have different effects on women [9, 10]. More insight into the impact of IPV on the well-being of mothers is needed [11]. Specifically, mothers have different challenges and social experiences along with higher rates of IPV [12], which increases their risk of poor health compared to women who do not face similar challenges [13, 14]. Mothers in developing countries encounter poor structural and living conditions. They often encounter sources of stressors related to poor mental and physical health. However, the health outcomes of abused women are different in diverse ethnic groups.

The majority of research on women ’ challenges to IPV screening has been undertaken in Western countries, which have a distinct cultural backdrop than Jordan’s Middle Eastern, conservative society. Although the frequency of IPV in Jordan is significant, requiring IPV screening, no research measuring the rate of IPV screening by Jordanian women or impediments to IPV screening were found. In addition, few Jordanian studies compared screening rates of women based on their personal IPV.

The investigation of the association between IPV and the quality of life of mothers in Jordan is particularly significant due to the country’s unique social and cultural, legal, and economic environment [9]. Here are some specific reasons why such a study is necessary in Jordan. Firstly, Jordan’s cultural and social environment is numerous and complicated. Understanding how IPV impacts mothers’s quality of life in Jordan enables academics and policymakers to adjust solutions to the population’s individual needs and cultural sensitivity [10]. Jordanian women also thought that victims’ actions—such as violating cultural and conventional expectations in marriage—could lead to IPV. Studies conducted that showed that Jordanian women faulted themselves for the abuse they experienced. Jordanian culture considered it imperative that traditional male-dominated marital roles be followed; to do otherwise was to violate social norms and established familial regulations, which could lead to intimate partner violence (IPV) [11, 12]. Thus, following this thinking system could account for the fact that over 60% of Jordanian men and women said that males should mistreat their wives in order to establish their power. Thus, following this belief system might be responsible for the fact that over 60% of Jordanian men and women justified mistreating women and asserting men’s dominance through spousal violence [13]. Moreover, a few Jordanian nurses thought that staying in an abusive relationship could have some benefits for the victims. According to Oweis et al. [15], financial reliance, a lack of family support, and the fear of losing custody of their children are the main reasons why Jordanian women may become victims of abuse. In spite of their cultural and personal beliefs regarding marriage, nurses are expected to protect victims and give them the proper care. Being Jordanians, these nurses held views consistent with Jordanian cultural norms, which may have contributed to their negative Moreover, Jordan has achieved significant progress in establishing legislative changes to address gender-based violence, including IPV. Investigating the association between IPV and quality of life may be helpful in evaluating the efficacy of these interventions [10, 11]. Such as numerous other nations, Jordan is dealing with IPV-related health and mental health issues. In this setting, research may be helpful in identifying the particular health and well-being consequences faced by Jordanian women, as well as informing healthcare policy and services [11]. Jordanian society puts an importance on family dynamics and child well-being. The study of the connection between IPV and mothers’s quality of life can give information on how IPV affects family structures and children’s lives. Moreover, community and religious leaders in Jordan are frequently influential in tackling societal issues. The findings of the study can be used to engage these leaders in public awareness campaigns and to promote efforts to prevent IPV [10]. It worth to mention there is no data about the quality of life for mothers during COVID-19, which make it important to have preliminary data about this issue.

This study provides more information to the already available research and literature demonstrating the harmful impacts of IPV by identifying the relationship between HRQoL performance and IPV among mothers in Jordan. The research findings reveal that IPV causes and worsens behavioral, financial, and mental health issues, which can disrupt HRQoL [15, 16]. Social workers and healthcare providers can use the identified results of this study to determine the most effective intervention to alleviate IPV impacts. The study aims to examine the correlation between IPV and HRQoL of mothers during COVID-19 era. Moreover, the correlates of IPV among mothers in Jordan were identified.

## Method

### Design

This is a cross-sectional correlational study that used a survey to assess the correlation between IPV and HRQoL for women in Jordan.

### Data collection

For survey creation, the authors make an outline of the survey that correspond with the goals of the study, then, they assured every item of the study is precise and concise and they used a variety of question formats, such as o Likert scale, multiple-choice, and demographics inquiries. To make the survey easier to navigate, the authors combine items together and employ logical flow. In order avoid participant fatigue, the authors kept the survey as brief as feasible. An informed consent statement that outlines the study’s goal, its private nature, and how the results was used. The survey with a small sample of women was piloted before distributing it to determine if there are any problems with the questions or the way it flows. The survey link was provided to women via social media websites such as WhatsApp, twitters and others with an online invitation letter outlining the study’s goal, voluntary participation, confidentiality, survey length, and potential advantages and dangers. In addition, an online consent form was created, and participants were urged to carefully read the informed consent and click on the agreement button at the bottom of the consent form if they were ready to participate in the study. The anonymity of the participants was ensured by not adding identifying details to the study survey. They were also not recognized by the e-survey software once the survey finished. Respondents should be aware that the study instruments may contain items that have the potential to trigger negative feelings. The study sample included mothers After identification of confidence interval (CI) of 95%, the statistical power of 80%, and α of 0.05 was used to select at least 280 mothers. To avoid missing of the data, the authors were collected 300 responses. The inclusion criteria was Jordanian mothers with at least 18 years old, who read and write in Arabic language and able to participate.

The Women Abusing Screening Tool (WAST) is an eight-item questionnaire that was given to samples of Arabic women [17]. Women responded to these items using a three-point scale. The answers were totaled to create an interval scale. For Arabic WAST, different scoring criteria were used. The scores are added together to form an interval scale. A cut-off score of 1 correctly classified 92 percent of victims and 100% of non-victims. To determine the victim status of domestic violence, a cut-off score of 2 is used, yielding a sensitivity of 89 percent and a specificity of 94 percent. Content validity of the Arabic version was done A. Al-Natour et al.. The nine items from the WAST were measured with a 3-point Likert scale (often, sometimes, never). The first two items revealed the experience of marital violence. This instrument was used in Arabic language with one of this paper authors, so the reliability and validity were checked. The Cronbach alpha .85.

The second instrument is for measuring quality of life (SF-36) Standard is made up of 35 items was divided into 8 subscales: (1) physical function (PF), limitations due to physical health problems (role physical, RP), (3) bodily pain (BP), (4) general health (GH), (5) vitality [VT], (6) social functioning (SF), (7) limitations due to emotional health problems (role emotional, RE), and (8) mental health. Each domain was assigned an SF-36 score between 0 and 100, with higher scores indicating better functional status [18]. The eight subdomain scores were combined into two summary measures: physical component summary (PCS) and mental component summary (MCS), with a low MCS or PCS with score of 50 indicating poor HRQoL. The Arabic version of SF-36 V2 was proved to be a valid instrument [Cronbach’s alpha >0.70] [19]. The twelve items of SF-36 V2 Physical wellbeing and mental health were measured with a 5-point Likert scale. Questions about physical wellbeing are scored on a scale from 0 to 100. A score of 100 represents the highest level of functioning. This instrument was used in Arabic language with one of this paper authors, so the reliability and validity were checked. The Cronbach alpha .85.

### Ethical consideration

This study was approved by Jordan University of Science and Technology (#777/2023). The authors were explained objective, benefits, harms, and all issues that related to the study on the cover sheet that was attached to each electronic link that was sent to the participant. The data was anonymous since no identified data was asked and the participants had the opportunity to withdraw at any time. The written consent form was signed electronically from all participants. The authors checked the instrument for reliability and validity. Moreover, the authors monitored the study in all steps from the idea to the completion of the study. The authors were assuring confidentiality by allowing only the main authors to get an access to the data. There are also password on the data file, so no one will have an access without password.

### Data analysis

SPSS In comparison to women who had not encountered IPV, women who had experienced partner abuse reported poorer HRQoL in all domains version 26 was used to analyze the data. Descriptive statistics was used for description of the sample characteristics and Pearson correlation was used for correlation between IPV and HRQoL. Before conducting Pearson correlation and multiple regression, the authors make sure that the variables that intend to correlate are both continuous, numerical variables. Also, they checked for linearity between the variables. Then, the authors assessed the normality of the variables to confirm that your data is reasonably normally distributed. Also, they examined the scatterplot for homoscedasticity, which means that the spread of data points should be roughly consistent across all values of the variable. Outliers: Identify and deal with outliers. Outliers can unduly influence the correlation coefficient. They can visually identify outliers using scatterplots and apply statistical tests to confirm their presence.

Multiple regressions test was used to determine the factors that impact violence toward IPV and HRQoL of multiple regression. The assumptions were evaluated since the assumptions can lead to misleading regression analysis results. The researcher performed a test to check for the presence of the four multicollinearities, which indicate whether the independent variable has high collinearity. All the variance inflation factor (VIF) values were < 4. 0, so they were below the maximum acceptable value (*VIF = 10*). This result indicated that the multicollinearity problem was insignificant. The authors recorded the items of categorical variables. For example, use male = 1 and female = 2.

## Results

### Demographic characteristics

The number of participants were 300 women. The mean age of the mothers is 30±5. More than 50% of women hold a bachelor’s degree. See Table 1.

**Table 1:** Demographic of participants in Jordan (N = 300).

Variable	Category	N	%
**Nature of Work**	Governmental	106	35.3
	Private	51	17.0
	Not working	131	43.7
	Student	12	4.0
Husband Work	Governmental	114	38.0
	Private	150	50.0
	Not working	36	12.0
**Living area**	City	255	85.0
	Village	45	15.0
**Medical insurance**	No	45	15.0
	Yes	255	85.0
**Smoking**	No	255	85.0
	Yes	45	15.0
**How many times hearing news**	Never	29	9.7
	Rarely	84	28.0
	Sometimes	85	28.3
	Usually,	102	34.0
**Degree**	Primary or secondary	25	8.3
	Associate	48	16.0
	Bachelor	150	50.0
	Graduate	77	25.7
**Husband Education**	Primary or secondary	62	20.7
	Associate	44	14.7
	Bachelor	194	64.7
**Household Income [JDs]**	Less than 400	68	22.7
	400 to 600	56	18.7
	600 to 800	54	18.0
	More than 800	122	40.7

### Correlation between IPV and HRQoL among mothers during COVID era

The prevalence of IPV among women was 28.3. The mean of quality of life is 86.0 (*SD = 13*.*1)* and the mean of violence is 11.9 (*SD = 3*.*01)*. There was a negative relationship between violence and quality of life (*x*^*2*^ *=* .*224*, *p =* .*001*). This means as the violence increases, the quality-of-life decreases.

### Correlates of quality of life among mothers during COVID-19

The model of regression was significant (*F = 9*.*64*, *p =* .*011*). This means many correlates predicted quality of life among mothers in Jordan. These factors that significant and impacted the model were working or not (*B = -*.*144*, *P =* .*031*) and income (*B =* .*223*, *p =* .*001*). Women who worked and had higher income showed higher quality of life compared to other mothers. See Table 2.

**Table 2:** Predictors of HRQoL among mothers.

Variables	Beta	t-test	P value	Lower CI	Upper CI
(Constant)	-.111	8.888	.000	71.766	112.59
Number of daughters	.056	-1.691	.092	-2.575	.196
Number of sons	.026	.855	.394	-.737	1.868
Nationality	-.144	.446	.656	-5.458	8.651
Working or not	.078	-2.163	.031	-7.126	-.335
Husband work	.056	1.216	.225	-.953	4.028
Marriage years	-.223	.711	.477	-1.647	3.510
Household Income	.026	-3.256	.001	-3.839	-.946
Living area(Rural or Urban)	-.018	.432	.666	-3.411	5.329
Type of education	-.033	-.244	.807	-2.494	1.944
Type of husband education	-.011	-.468	.640	-2.757	1.698
Do you smoke?	.011	-.189	.850	-4.701	3.876
Do you have health medical insurance?	-.045	.171	.865	-4.174	4.967
How many times do you hear the news?	-.111	-.757	.450	-2.122	.944

The model of regression was significant (*F = 9*.*64*, *p =* .*011*). This means many correlates were predicted IPV among mothers in Jordan. These factors that significant and impact the model were marriage years (*B =* .*181*, *P =* .*015*), type of working for the husband (*B =* .*157*, *P =* .*021*) and income (*B =* .*165*, *p =* .*013*). Other demographics and factors such as living area and number of children were found to be not significant and correlated with IPV. Women who had increased years of marriage, and not had work and had lower income showed to be suffered from more IPV compared to other mothers. See Table 3.

**Table 3:** Predictors of IPV among mothers.

Variables	Beta	t-test	P value	Lower CI	Upper CI
Constant		4.943	0	6.755	15.696
Number of females	0.005	0.072	0.942	-0.295	0.318
Number of males	-0.057	-0.915	0.361	-0.416	0.152
Nationality	-0.003	-0.057	0.954	-1.608	1.517
Type of work	-0.061	-0.973	0.331	-1.113	0.377
Husband work	0.043	0.697	0.486	-0.354	0.741
Number of marriage years	0.181	2.451	0.015	0.137	1.254
Household Income	-0.165	-2.51	0.013	-0.73	-0.088
Living area	-0.007	-0.128	0.898	-1.029	0.903
Type of education	0.01	0.14	0.889	-0.455	0.524
Type of husband education	-0.157	-2.317	0.021	-1.064	-0.087
Do you smoke?	0.06	1.067	0.287	-0.428	1.44
Do you have medical insurance?	-0.029	-0.469	0.64	-1.253	0.771
How many times do you hear the news?	-0.048	-0.84	0.402	-0.481	0.193

## Discussion

The IPV is a dangerous form of violence that has a significant impact on women’s health and Jordanian women were not an exception. The overall prevalence of any IPV among mothers in this study was 28%. Similarly, in Tadesse et al [20] the IPV among women was 22%. In Bangladesh, 56% of women in rural regions and 48.7% of women in urban areas say their husband has abused them physically or sexually [21]. Hidden IPV prompted by financial difficulty were the most prevalent reasons mentioned by women, whereas males named their spouses’ disobedience as the primary factor. Stress from losing work and income could be a precipitating factor among men during the epidemic. Our findings on the prevalence of IPV were consistent with those of studies undertaken in Ontario and the United States [22]. This could be due to comparable population features in terms of how they respond to psychological or financial stress when at home.

Our study found there was a negative relationship between violence and quality of life (r^2^ = .224, p = .001). This means as the violence increases, the quality of life is decreased. Many studies found that in n comparison to women who had not encountered IPV, women who had experienced partner abuse reported poorer HRQoL in all domains [23]. Women seeking help for intimate partner abuse had particularly low mental health scores. New research from Iran, China, and the United States confirms that battered women have much lower quality-of-life scores than non-abused women [23–25]. Domestic abuse among pregnant women is inversely connected with health-related quality of life in Iran, according to Tavoli et al. [23] and Gharacheh et al. [24]. Bedford et al. [25] found that in China, women who experienced sexual assault from their spouse had significantly inferior health-related quality of life.

Our study found that mothers with greater years of marriage, and not having work and had lower income showed more suffering from IPV compared to other participants in the study. The only correlates of violence among women, according to Abuhammad [10], were not having a job (p = .001) and marital status (p = .001). During the COVID-19 pandemic, many women found themselves jobless. It was discovered that unemployed women are more likely to be victims of violence than women who earn a higher wage or have another source of income [26]. Since these women were dependent to the husband, this will impose her to more types of violence. In addition, the amount of time spent in close touch with a spouse or family member grew, causing tension and conflicts. Age is not a correlate of aggression, according to our findings. However, according to a few studies, many older battered women do not report the violence or its repercussions to the authorities. The likelihood of aggression was raised by a history of alcohol and drug abuse. Many studies found that women with a husband with low education experience IPV compared to other mothers [15, 26–28]. It worth to mention that women from Middle Eastern cultures remained in abusive marriages during times of war for a variety of reasons, including culturally endorsed notions of male dominance and superiority, a lack of parental support to end the marriage, financial dependence on the men, stigmatization by society of divorced women, and concern over losing custody of their children [26]. The women in this survey confirmed these sentiments. Men are granted superiority over women in Middle Eastern societies [15, 26], including the right to chastise both wife and children. Due to Jordan’s acceptance of this practice and its patriarchal society, it is extremely difficult for women to leave an abusive relationship without experiencing exclusion and discrimination [26]. Other conservative civilizations in the Middle East and Africa likewise exhibit male dominance and the consequent subordination of women [28].

Our study found that women who worked and had higher income during COVID-19 era showed higher quality of life compared to other women in the study. The individuals’ degree of education and employment status are other important correlates to HRQoL for mothers. Evidence suggests that low IPV relates to education level and income among mothers [29, 30]. According to the report, rural residents were three times more likely than their urban counterparts to contract IPV. This could be due to the low socioeconomic status of people who live in villages. Furthermore, because there is a dearth of information about how to deal with violence. Studies conducted in several parts of Ethiopia corroborated this conclusion [31, 32].

### Implication for the study

The result of this study may help Jordan’s National Strategy for Combating Violence Against Women to make steps toward combating gender-based violence. Also, evidence from research may be utilized to shape policy and argue for change. Moreover, understanding the particular risk factors and barriers in Jordan may help in the development of culturally responsive preventative and intervention strategies since Jordan signed several international human rights agreements. Research can enable the country’s efforts to protect women’s rights and eradicate violence against women to be more in line with its global obligations.

### Limitations

Because the data is cross-sectional, it is impossible to assess causal correlations between research variables. Because the poll was performed online, the authors were only able to reach out to those who may be most vulnerable to IPV. It is critical to recognize that the data has limited generalizability. The women from one urban region, most of them were white. Because it is unknown if the emotional and psychological consequences of violence vary depending on geographic location, our findings must be interpreted with caution. This study was talking about IPV without asking about different categories of it. Moreover, this study used self-report bias that may lead to underreporting of abuse and its consequences; thus, our findings may be considered as a lower limit of the effect. Moreover, many women with low quality of life have no access to the internet and could not take the survey.

## Conclusion

The study showed there is a negative relationship between IPV and HRQoL during COVID-19 era. Women who worked and had higher income showed higher quality of life compared to other mothers. Furthermore, Women who had increased years of marriage, and not had work and had lower income showed more violence compared to other mothers. It is critical to test mothers for domestic violence at these and other relevant centers, as well as to implement appropriate programs to combat IPV to enhance the quality of life for abused women. Thus, healthcare officials and specialists involved in women’s care, particularly during terrible, crisis situations like the COVID-19 epidemic, should be informed of the scope of the problem and take appropriate steps to address it. Successfully implementing measures to prevent and mitigate violence against women is critical.

## Supporting information

S1 Data (XLSX)

S1 File (SAV)
